# Human *N*-Acetyltransferase 1 and 2 Differ in Affinity Towards Acetyl-Coenzyme A Cofactor and *N*-Hydroxy-Arylamine Carcinogens

**DOI:** 10.3389/fphar.2022.821133

**Published:** 2022-02-25

**Authors:** David W. Hein, Mark A. Doll, Mariam R. Habil

**Affiliations:** Department of Pharmacology and Toxicology, University of Louisville School of Medicine, Louisville, KY, United States

**Keywords:** acetyl coenzyme A, arylamine *N*-acetyltransferase 1, arylamine N-acetyltransferase 2, *N*-acetylation, *O*-acetylation, affinity

## Abstract

Arylamine N-acetyltransferases catalyze the transfer of acetyl groups from the endogenous cofactor acetyl coenzyme A (AcCoA) to arylamine (*N*-acetylation) and *N*-hydroxy-arylamine (*O*-acetylation) acceptors. Humans express two arylamine *N*-acetyltransferase isozymes (NAT1 and NAT2) which catalyze both *N*- and *O*-acetylation but differ in genetic regulation, substrate selectivity, and expression in human tissues. We investigated recombinant human *NAT1* and *NAT2* expressed in an *Escherichia coli* JM105 and *Schizosaccharomyces pombe* expression systems as well as in Chinese hamster ovary (CHO) cells to assess the relative affinity of AcCoA for human NAT1 and NAT2. NAT1 and NAT2 affinity for AcCoA was higher for recombinant human NAT1 than NAT2 when catalyzing *N*-acetylation of aromatic amine carcinogens 2-aminofluroene (AF), 4-aminobiphenyl (ABP), and β-naphthylamine (BNA) and the metabolic activation of *N*-hydroxy-2-aminofluorene (*N*-OH-AF) and *N*-hydroxy-4-aminobiphenyl (*N*-OH-ABP) via *O*-acetylation. These results suggest that AcCoA level may influence differential rates of arylamine carcinogen metabolism catalyzed by NAT1 and NAT2 in human tissues. Affinity was higher for NAT2 than for NAT1 using *N*-OH-AF and *N*-OH-ABP as substrate consistent with a larger active site for NAT2. In conclusion, following recombinant expression in bacteria, yeast, and CHO cells, we report significant differences in affinity between human NAT1 and NAT2 for its required co-factor AcCoA, as well as for *N*-hydroxy-arylamines activated via *O*-acetylation. The findings provide important information to understand the relative contribution of human NAT1 vs NAT2 towards *N*-acetylation and *O*-acetylation reactions in human hepatic and extrahepatic tissues.

## Introduction

*N*-acetyltransferase 1 (NAT1) and 2 (NAT2) catalyze the *N*-acetylation of carcinogenic arylamines. Following *N*-hydroxylation by cytochrome P450s, NAT1 and NAT2 catalyze the *O*-acetylation of their *N*-hydroxylated metabolites to unstable *N*-acetoxy metabolites which bind to DNA leading to mutagenesis and carcinogenesis (Windmill et al., 1997; Wang et al., 2019). Genetic polymorphisms in NAT1 or NAT2 are associated with increased cancer risk at numerous sites (reviewed in Hein et al., 2000; Agundez, 2008) including urinary bladder (Garcia-Closas et al., 2005) and head and neck (Mohammadi et al., 2021) cancers.

*NAT1* and *NAT2* open reading frames are 87% identical and their proteins differ only in 55 amino acids (Hein et al., 2000). The crystal structures, three-dimensional modeling, and docking simulations show that the substrate binding pocket in NAT1 is smaller than that of NAT2 as a consequence of amino acid residue substitutions at positions 127 and 129, namely R127 and Y129 in NAT1 as opposed to S127 and S129 in NAT2 (Wu et al., 2007; Zhou et al., 2013). The two bulkier amino acids reduce the volume of the NAT1 pocket by ∼40% compared to NAT2. In addition, a change from V93 in NAT1 to F93 in NAT2 introduces a bump in the van der Waals surface of the pocket in NAT2, thereby significantly altering the shape of the binding pocket likely contributing to substrate specificity (Wu et al., 2007; Zhou et al., 2013). For example, NAT1 shows substrate specificity for *p*-aminobenzoic acid while NAT2 shows substrate specificity for sulfamethazine (Doll et al., 2010). Whereas previous studies have investigated the substrate specificity of human NAT1 and NAT2 for *N*-acetylation (Hein et al., 1993; Leggett et al., 2021), no study to our knowledge has investigated NAT1 and NAT2 affinity for the *O*-acetylation of *N-*hydroxy-arylamines.

Metabolic activation of *N*-OH-AF and *N*-OH-ABP via *O*-acetylation is catalyzed by recombinant human NAT1 and NAT2 expressed in bacteria (Hein et al., 1993, Hein et al., 1995; Doll and Hein, 2017), yeast (Fretland et al., 2002), and both COS-1 (Zang et al., 2007; Zhu et al., 2011) and Chinese hamster ovary (CHO) (Millner et al., 2012; Baldauf et al., 2020) cells. *N*-OH-ABP metabolic activation via *O*-acetylation also has been reported in cryopreserved human hepatocytes (Doll et al., 2010). CHO cells expressing *CYP1A2* and rapid acetylator NAT2*4 exhibit greater DNA adducts and mutations than CHO cells expressing *CYP1A2* and slow acetylator NAT2*5B following incubations with low concentrations of 2-aminofluorene (AF) and 4-aminobiphenyl (ABP) (Baldauf et al., 2020) suggesting an important role of NAT2-catalyzed *O*-acetylation in the metabolic activation of arylamine carcinogens in tissues expressing NAT2.

AcCoA binds to cysteine 68 (Rodrigues-Lima et al., 2001; Rodrigues-Lima and Dupret, 2002) in a catalytic triad of Cys-68, His-107, Asp-122 in both NAT1 and NAT2 (Sinclair et al., 2000). Previous determinations of human NAT1 and NAT2 affinity for AcCoA were conducted for recombinant NAT2 expressed in bacteria (Hein et al., 1993) and recombinant NAT1 expressed in yeast (Zhu and Hein, 2008) with substrates specific for NAT1- and NAT2-catalyzed *N*-acetylation. Arylamine carcinogens such as 2-aminofluorene (AF), 4-aminobiphenyl (ABP) and β -naphthylamine (BNA) undergo *N*-acetylation catalyzed by human NAT1 and NAT2 following recombinant expression in bacteria (Hein et al., 1993) or yeast (Leggett et al., 2021) and thus are more appropriate to use for comparing human NAT1 and NAT2 affinity for AcCoA.

Previous investigations (Minchin et al., 1992; Probst et al., 1992; Hein et al., 1993) reported that the *O*-acetylation of *N*-hydroxy-2-aminofluorene (*N*-OH-AF) and *N*-hydroxy-4-aminobiphenyl (*N*-OH-ABP) was catalyzed by both human NAT1 and NAT2. Although NAT1 “appeared” to be more selective for the N-hydroxy derivatives of carboxylic arylamine carcinogens (Minchin et al., 1992; Hein et al., 1993), this has not to our knowledge been the focus of a more robust investigation comparing their substrate affinities for human NAT1 and NAT2.

## Materials and Methods

### Expression of Recombinant Human *N*-Acetyltransferase 1 and 2 in Bacteria

Recombinant expression of human NAT1*4 and NAT2*4 (the reference or “wild-type” human NAT1 and NAT2 alleles) in bacteria was performed as previously described (Hein et al., 1993). Briefly, JM105 bacteria harboring human NAT1*4 or NAT2*4 plasmids were prepared and grown up overnight in Luria-Bertani (LB) medium containing 100 μg/ml ampicillin (LB-Amp) at 37°C. Fresh LB-Amp broth was re-inoculated and NAT1- and NAT2-expression bacteria and grown to approximately 0.5 OD_600nm_. Isopropyl β -D-thiogalactopyranoside (1 mM) was added to the broth for induction, and the cultures were grown for an additional 3 h. The cells were harvested by centrifugation, then resuspended in 20 mM sodium phosphate buffer, pH 7.4, containing 1 mM EDTA and DTT, 10 µM leupeptin and 100 µM phenylmethylsulfonyl fluoride to 5% of original culture volume. The cells were sonicated for 6 × 30 s on ice. Lysates were centrifuged to pellet bacterial debris. Protein concentrations of bacterial lysates were determined by a Bio-Rad dye-binding method (Bradford, 1976). Enzyme velocities were then normalized relative to the quantity [arbitrary units (U)] of immunoreactive NAT1 or NAT2 protein detected by Western immunoblot analysis. Bacterial lysates (50 µg) containing NAT1 or NAT2 were mixed with SDS-polyacrylamide gel sample buffer containing 5% (final) β -mercaptoethanol and boiled for 5 min. The protein samples were separated on 12% SDS-polyacrylamide gels, electrophoretically transferred to Immuno-Lite membranes (Bio-Rad, Richmond, CA) and reacted to polyclonal rabbit antiserum raised against purified human NAT2 (kindly provided by Dr. Denis Grant, University of Toronto). Chemiluminescent detection was achieved with an Immuno-Lite kit (Bio-Rad, Richmond, CA) following manufacturer’s instructions as previously described (Zenser et al., 1996).

The lysates were assayed for AF *N*-acetyltransferase and *N*-hydroxy-AF or *N*-hydroxy-ABP *O*-acetyltransferase activities as described below.

### AF *N*-Acetyltransferase Assays

The *N*-acetylation of AF was determined by measuring 2-acetylaminofluorene product after separation by high performance liquid chromatography (HPLC) as previously described (Doll and Hein, 2017). The reaction mixture (100 µl) contained bacterial lysate and 100 µM AF and AcCoA. AF and AcCoA were purchased from Sigma Chemicals, St. Louis, MO. Duplicate reactions were performed at AcCoA concentrations ranging from 0.031 to 10 mM. Control reactions were conducted in the absence of AcCoA.

### *N-*OH-AF and *N*-Hydroxy-ABP *O*-Acetyltransferase Assays

AcCoA-dependent metabolic activation of [*ring*-^3^H]*N*-OH-AF and [*ring*-^3^H]*N*-OH-ABP (Chemsyn Science Laboratories, Inc (Lenexa, KS) to DNA adducts was conducted as previously described (Hein et al., 1993; Hein et al., 1995; Doll and Hein, 2017). The reaction mixture contained 20 mM sodium phosphate buffer (pH 7.4), 1 mM DTT, 1 mM EDTA, 100 µM [*ring*-^3^H]*N*-OH-AF or [*ring*-^3^H]*N*-OH-ABP, 1 mg/ml calf thymus DNA (Sigma Chemicals, St. Louis, MO), AcCoA and suitably diluted bacterial lysate. For determinations of *N*-OH-AF and *N*-OH-ABP affinity, concentrations of *N*-hydroxy substrate varied from 5 to 1,000 µM in the presence of 300 µM AcCoA except for *N*-OH-AF catalyzed by NAT2 where AcCoA concentration was 1,000 µM. For determination of AcCoA affinity, concentrations of AcCoA ranged from 0.006 to 10 mM in the presence of 100 μM *N*-OH-AF. Control reactions were conducted in the absence of AcCoA.

### Expression of Recombinant Human *N*-Acetyltransferase 1 and 2 in Yeast

Recombinant human NAT1 and NAT2 were stably expressed in yeast (*Schizosaccharomyces pombe*) as previously described (Fretland et al., 2001a; 2001b). Quantitation of specific human NAT1 and NAT2 protein in yeast lysates has been described (Fretland et al., 2001a; 2001b). *O*-acetyltransferase assays containing yeast lysate, AcCoA, 1 mg/ml deoxyguanosine (dG), and *N*-OH-AF or *N*-OH-ABP substrate were incubated at 37°C for 10 min as previously described (Hein et al., 2006a). *N*-OH-AF and *N*-OH-ABP were purchased from Toronto Research Chemicals, Toronto, Canada; dG was purchased from Sigma Chemicals, St. Louis, MO. Control reactions were conducted in the absence of AcCoA. For determination of apparent Km, *N*-hydroxy-arylamine concentrations ranged from 1.95 to 2,000 µM in the presence of 1 mM AcCoA. AcCoA concentrations ranged from 4.5 to 5,000 µM in the presence of 500 μM N-hydroxy-arylamine. HPLC separation was achieved using a gradient of 80:20 sodium perchlorate pH 2.5: acetonitrile to 50:50 sodium perchlorate pH 2.5: acetonitrile over 3 min and dG-C8-arylamine adduct was detected at 300 nm.

### Expression of Human *N*-Acetyltransferase 1 and 2 in Chinese Hamster Ovary Cells

To further investigate AcCoA affinity for human NAT1 and NAT2, we incorporated UV5-CHO cells that express human CYP1A1 and NAT1 (constructed to reflect extrahepatic metabolism) and human CYP1A2 and NAT2 (constructed to reflect hepatic metabolism). The CHO cells expressing CY1A1 or CYP1A2 and NAT1 or NAT2 were constructed previously to assess DNA damage and mutagenesis *in situ* following exposure to arylamine carcinogens. These CHO cells also were used to investigate the relative affinity of AcCoA for NAT1 and NAT2 *in vitro* as described below. The construction and characterization of CHO cells expressing human NAT1*4 with NAT1b promotor (Millner et al., 2012) and NAT2*4 (Metry et al., 2007) including quantitation of specific human NAT1 and NAT2 protein (Salazar-Gonzalez et al., 2020) has been described previously. Briefly, UV5/CHO cells were stably transfected with a single FRT integration site using Flp-In System from Invitrogen (Metry et al., 2007). The UV5/FRT cells was modified by stable integration of human *CYP1A1* and *NAT1* (Millner et al., 2012) *or CYP1A2* and *NAT2* (Metry et al., 2007). The *NAT1*-transfected cells were characterized for *N*-acetylation of *p*-aminobenzoic acid, a NAT1-selective substrate (Millner et al., 2012) and the NAT2-transfected cells were characterized for *N*-acetylation of sulfamethazine, a NAT2-selective substrate (Metry et al., 2007). ABP and BNA *N*-acetyltransferase assays on NAT1- and NAT2-transfected CHO cells were carried out as described below.

### ABP and BNA *N*-Acetyltransferase Assays

*N*-acetyltransferase assays containing CHO cell lysates expressing human NAT1 or NAT2, ABP (300 µM) or BNA (250 or 62.5 µM for NAT1 and NAT2 respectively) and AcCoA (31.3–5,000 µM) were incubated at 37°C for 60 min. ABP and BNA were purchased from Sigma Chemicals, St. Louis, MO. Reactions were terminated by the addition of 1/10 volume of 1 M acetic acid and the reaction tubes were centrifuged for 10 min to precipitate protein. The amount of acetyl-ABP produced was determined following separation and quantitation by high performance liquid chromatography as described previously (Habil et al., 2020). Control reactions were conducted in the absence of AcCoA. The amount of acetyl-BNA produced was determined following separation and quantitation by HPLC subjected to a gradient of 85% 20 mM sodium perchlorate pH 2.5/15% acetonitrile to 35% 20 mM sodium perchlorate pH 2.5/65% acetonitrile over 10 min, then to 85% 20 mM sodium perchlorate pH 2.5/15% acetonitrile over 5 min onto a 125 × 4 mm 100 RP-100 5 µM C18 column. Retention times for BNA and acetyl-BNA were 3.97 and 10.1 min, respectively. Absorbance was detected at 260 nm.

### Data Analysis

Protein concentrations were measured using the Bio-Rad assay kit (Hercules, CA, United States). Apparent Km values were calculated using the Michaelis-Menten program in Graphpad Prism software (San Diego, CA, United States) and differences in apparent Km between human NAT1 and NAT2 were tested for significance by unpaired t-test (2-tailed). Apparent Vmax values normalized to immunoreactive NAT protein were calculated following recombinant expression of human NAT1 and NAT2 in bacteria.

## Results and Discussion

**FIGURE 1:**
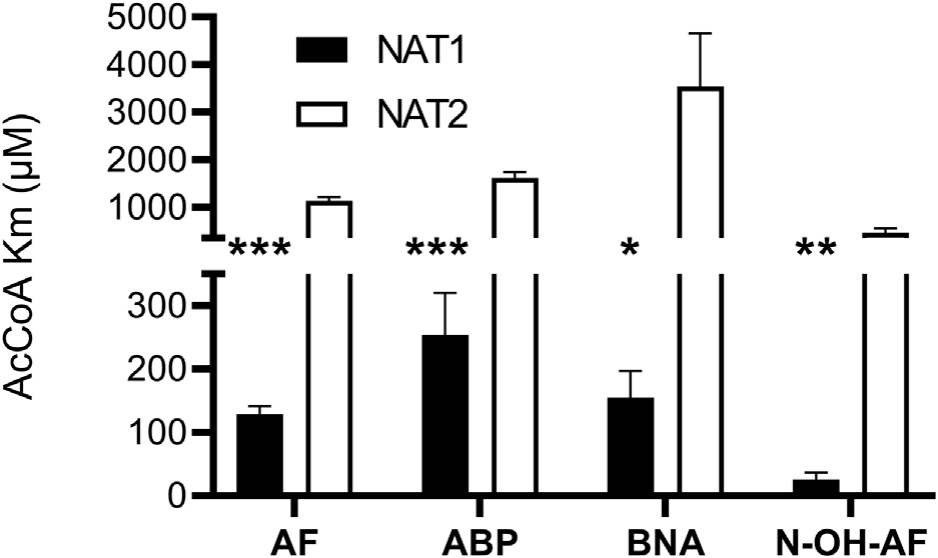
Relative affinity of AcCoA for human NAT1 and NAT2 expressed in bacteria or CHO cells. Each bar illustrates Mean ± SEM for three separate determinations of apparent AcCoA Km with substrates AF or *N*-OH-AF (expressed in bacteria) and ABP or BNA (expressed in CHO cells). AcCoA apparent Km was significantly lower *p < .05; **p < .01; ***p < .001 towards NAT1.

**FIGURE 2:**
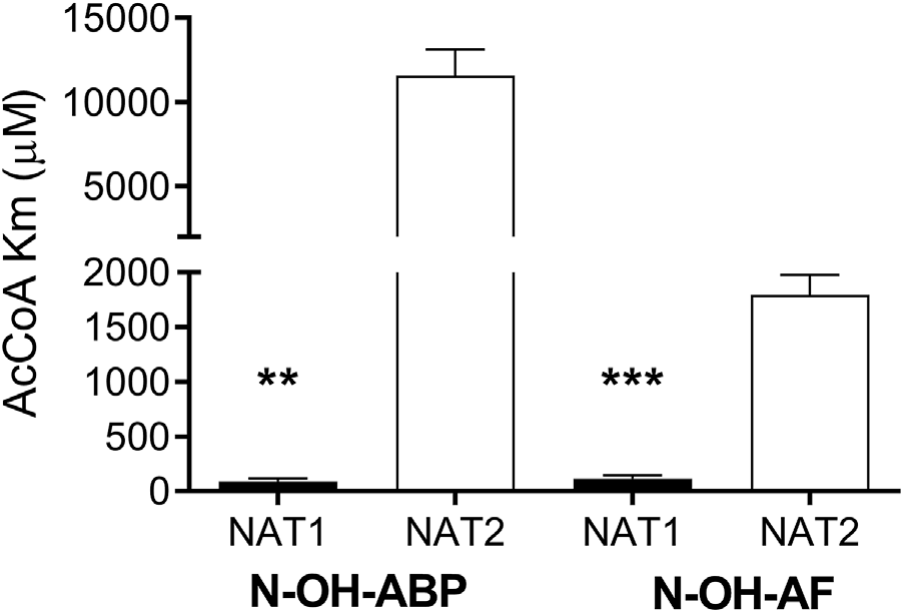
Relative affinity of AcCoA for human NAT1 and NAT2 recombinantly expressed in yeast. Each bar illustrates Mean ± SEM for three separate determinations of AcCoA Km in *N*-OH-ABP or *N*-OH-AF O-acetylation. AcCoA apparent Km was significantly lower **p < .01; ***p < .001 towards NAT1.

We investigated recombinant human *NAT1* and *NAT2* expressed in an *Escherichia coli* JM105 and *Schizosaccharomyces pombe* expression systems as well as in Chinese hamster ovary (CHO) cells to assess the relative affinity of AcCoA for human NAT1 and NAT2. NAT1 and NAT2 affinity for AcCoA was higher for recombinant human NAT1 than NAT2 when catalyzing *N*-acetylation of aromatic amine carcinogens AF, ABP, and BNA and the metabolic activation of *N*-hydroxy-AF and *N*-hydroxy-ABP via *O*-acetylation (Figure 1). Following recombinant expression of human NAT1 and NAT2 in yeast, the higher affinity of recombinant human NAT1 for AcCoA compared to human NAT2 when catalyzing the metabolic activation of *N*-hydroxy-ABP and *N*-hydroxy-AF *via O*-acetylation was confirmed (Figure 2). These results suggest that AcCoA level may influence differential rates of carcinogenic aromatic amine metabolism catalyzed by NAT1 and NAT2 in human tissues. In tissues where both NAT1 and NAT2 are expressed, low AcCoA levels may favor greater catalysis by NAT1 than NAT2.

AcCoA is an essential intermediate in diverse metabolic pathways, and cellular AcCoA levels fluctuate according to extracellular nutrient availability and the metabolic state of the cell (Shi and Tu, 2015). Thus, AcCoA levels are highly variable dependent upon several factors including fasted versus fed states. High AcCoA amounts occur in “growth” or “fed” states and promote its utilization for lipid synthesis and histone acetylation. In contrast, under “survival” or “fasted” states, AcCoA levels are lower because it is preferentially directed into the mitochondria to promote mitochondrial-dependent activities such as the synthesis of ATP and ketone bodies. Many methods used to measure AcCoA are inaccurate in part due to its instability (Sidoli et al., 2019). AcCoA levels have been reported in hepatic and extrahepatic tissues (Shurubor et al., 2020) and in human breast cancer cells (Stepp et al., 2019) on a per cell basis or on a per unit of protein which is not directly applicable to the Km values reported in our study. Cellular AcCoA concentrations of approximately 20–200 µM has been reported (Henry et al., 2015) which is within the range of NAT1 AcCoA Km but is much lower than the NAT2 AcCoA Km determined in our study. It also should be emphasized that the AcCoA Km determined *in vitro* in our study is an apparent Km dependent upon the concentration of the co-substrate N-hydroxy-arylamine. Nevertheless, as may be the case with the acetylation of histones, the *O*-acetylation of *N*-hydroxy-arylamine carcinogens catalyzed by NAT2 may be restricted by availability of AcCoA. When AcCoA levels rise or fall, it may modify the relative contribution of NAT1 versus NAT2 towards arylamine carcinogen metabolism in tissues in which both NAT1 and NAT2 are expressed.

NAT1 and NAT2 Vmax were not compared following recombinant expression in bacteria, COS-1 cells or CHO cells as the results are determined by the expression system which likely differs between NAT1 and NAT2. Although, outside of scope of the present study, measurement of NAT1 and NAT2 Vmax in human tissues would be much more relevant and would surely vary considerably between different human tissues, as has been shown for catalytic activities. For example, in the liver where NAT2 is highly expressed and NAT1 is not, acetylation will be catalyzed primarily by NAT2 despite the lower AcCoA Km for NAT1. However, in extrahepatic tissues where NAT1 is highly expressed and NAT2 is not, acetylation will be catalyzed primarily by NAT1, and this selectively is enhanced further by the higher affinity of NAT1 than NAT2 for AcCoA.

Previous studies showed that acetylation of the active site cysteine in NAT1 protects it from proteosomal degradation (Butcher et al., 2004) and that NAT1 but not NAT2 catalyzes hydrolysis of acetyl CoA to form acetyl and CoA in a folate-dependent manner (Laurier et al., 2014; Stepp et al., 2015). The apparent AcCoA Km for hydrolysis catalyzed by recombinant human NAT1 was reported as 54.3 µM (Stepp et al., 2015) which falls within the range of AcCoA apparent Km values determined with human recombinant NAT1 towards arylamine and *N*-hydroxy-arylamine carcinogens following recombinant expression from bacteria (Figure 1) and yeast (Figure 2). A previous investigation (Zhu and Hein, 2008) of AcCoA apparent Km for recombinant NAT1 expressed from COS-1 cells was determined at a high concentration (750 µM) of the aromatic amine substrate p-aminobenzoic acid, which likely resulted in elevated apparent AcCoA Km.

Following publication of high-resolution crystal structures of human NAT1 and NAT2 (Wu et al., 2007) three amino acid differences were identified for CoA binding (Zhou et al., 2013) that may be a factor for our results showing higher AcCoA affinity for NAT1 than NAT2. The N6 of the coenzyme A adenine ring forms a single hydrogen bond with serine-287 in NAT2 which is changed to phenylalanine-287 in NAT1. Leucine-288 generates van der Waals contacts with the pantothenate moiety of CoA but this is changed to phenylalanine-288 in NAT1. The carbonyl group of the pantothenate moiety establishes a hydrogen bond with serine-216 in NAT1 which is changed to valine-216 in NAT1.

**FIGURE 3:**
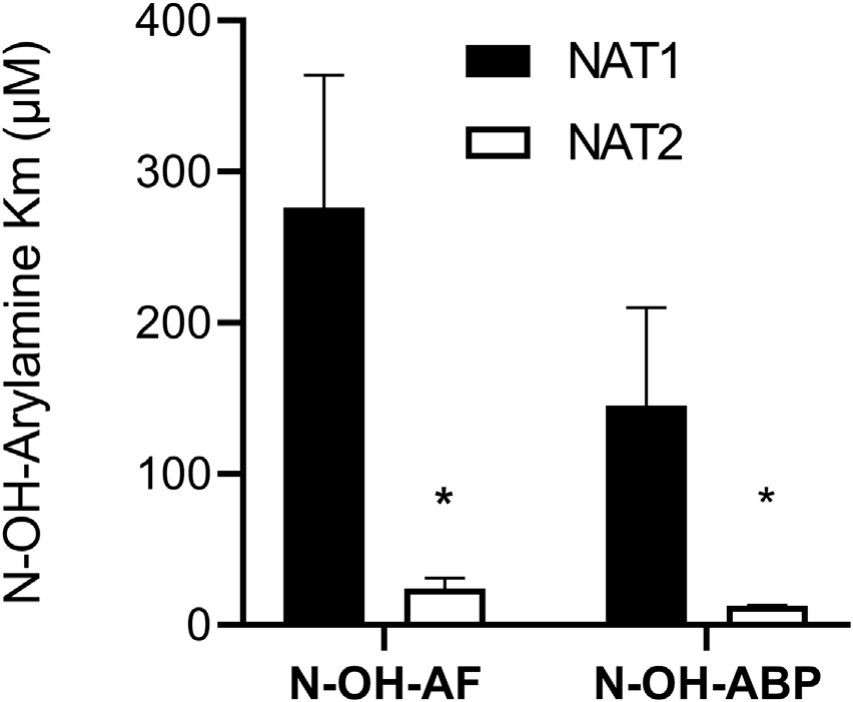
Relative affinity of *N*-OH-AF or *N*-OH-ABP for human NAT1 and NAT2 recombinantly expressed in bacteria. Each bar illustrates Mean ± SEM for three separate determinations. *NAT2 apparent Km for *N*-hydroxy-AF and *N*-hydroxy-ABP significantly lower (*p* < .05) than NAT1.

**FIGURE 4:**
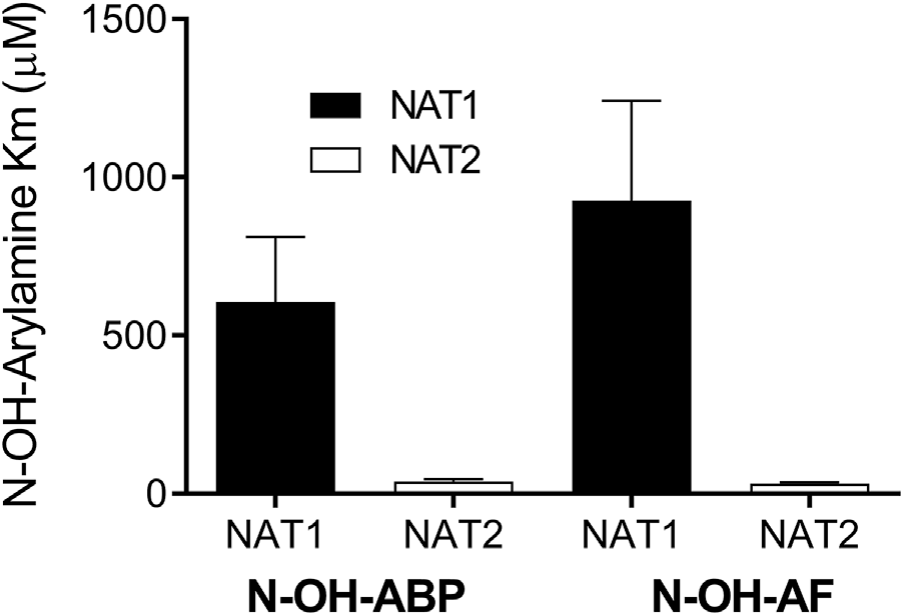
Relative affinity of human NAT1 and NAT2 for *N*-OH-AF or *N*-OH-ABP following recombinant expression in yeast. Each bar illustrates Mean ± SEM for three separate determinations. NAT1 apparent Km higher than NAT2 for *N*-OH-ABP (*p* = .0521) and *N*-OH-AF (*p* = .0474).

Recombinant human NAT1 and NAT2 affinity for *N*-OH-AF and *N*-OH-ABP was higher for human NAT2 than for NAT1 following recombinant expression from bacteria (Figure 3) or yeast (Figure 4). This finding is consistent with the 40% larger size of the active site for NAT2 (Wu et al., 2007). Indeed, the smaller NAT1 active site precludes the *O*-acetylation of *N*-hydroxy-heterocyclic amines (Minchin et al., 1992; Probst et al., 1992; Hein et al., 2006b; Lau et al., 2006). Previous studies showed higher affinity of recombinant human NAT2 than NAT1 for arylamine carcinogens (Hein et al., 1993). Our findings are consistent with the NAT2-genotype-dependent *O*-acetylation of *N*-OH-ABP observed in cryopreserved human hepatocytes (Doll et al., 2010) in which both NAT1 and NAT2 are expressed. The higher affinity of *N*-OH-AF and *N*-OH-ABP for human NAT2 also is consistent with recent findings in which CHO cells expressing *CYP1A2* and rapid acetylator NAT2*4 experienced greater DNA adducts and mutations than CHO cells expressing *CYP1A2* and slow acetylator NAT2*5B following incubations with low concentrations of AF and ABP (Baldauf et al., 2020).

In conclusion, following recombinant expression in bacteria, yeast, and CHO cells, we report significant differences in affinity between human NAT1 and NAT2 for its required co-factor AcCoA, as well as for the *O*-acetylation of *N*-hydroxy-arylamines. The findings provide important information to understand the relative contribution of human NAT1 vs NAT2 towards *N*-acetylation and *O*-acetylation reactions in human hepatic and extrahepatic tissues. In addition to xenobiotic metabolism, the present work may bring novel perspectives for the Phase II drug-metabolizing enzyme NAT1 and NAT2 in the metabolism of small molecule drugs.

## Data Availability

The original contributions presented in the study are included in the article/supplementary material, further inquiries can be directed to the corresponding author.
